# Plasma levels of microRNA-24, microRNA-320a, and microRNA-423-5p are potential biomarkers for colorectal carcinoma

**DOI:** 10.1186/s13046-015-0198-6

**Published:** 2015-08-22

**Authors:** Zanxi Fang, Jing Tang, Yongying Bai, Huayue Lin, Hanyu You, Hongwei Jin, Lingqing Lin, Pan You, Juan Li, Zhang Dai, Xianming Liang, Yuanhui Su, Qing Hu, Fen Wang, Zhong-Ying Zhang

**Affiliations:** Center for Clinical Laboratory, Xiamen University Affiliated Zhongshan Hospital, Xiamen, China; General Hospital of the Yangtze River Shipping, Wuhan, China; State Key Laboratory of Molecular Vaccinology and Molecular Diagnostics, School of Public Health, Xiamen University, Xiamen, China; Center for Cancer and Stem Cell Biology, Institute of Biosciences and Technology, Texas A&M Health Science Center, Houston, TX USA

**Keywords:** microRNA, Plasma, Colorectal cancer, Biomarker, Diagnosis

## Abstract

**Background:**

MicroRNAs are stable and easy to detect in plasma. The plasma levels of microRNAs are often changed in disease conditions, including cancer. This makes circulating microRNAs a novel class of biomarkers for cancer diagnosis. Analyses of online microRNA data base revealed that expression level of three microRNAs, microRNA-24 (miR-24), microRNA-320a (miR-320a), and microRNA-423-5p (miR-423-5p) were down-regulated in colorectal cancer (CRC). However, whether the plasma level of these three microRNAs can serve as biomarkers for CRC diagnosis and prognosis is not determined.

**Methods:**

Plasma samples from 223 patients with colorectal related diseases (111 cancer carcinoma, 59 adenoma, 24 colorectal polyps and 29 inflammatory bowel disease) and 130 healthy controls were collected and subjected to reverse transcription-quantitative real time PCR (RT-qPCR) analyses for the three microRNAs. In addition, plasma samples from 43 patients were collected before and after surgical treatment for the same RT-qPCR analyses.

**Results:**

The concentrations of plasma miR-24, miR-320a and miR-423-5p were all decreased in patients with CRC and benign lesions (polyps and adenoma) compared with healthy controls, but increased in inflammatory bowel disease (IBD). The sensitivity of miR-24, miR-320a and miR-423-5p for early stage of CRC were 77.78 %, 90.74 %, and 88.89 %, respectively. Moreover, the plasma concentration of the three microRNAs was increased in patients after the surgery who had clinical improvement.

**Conclusions:**

The plasma levels of miR-24, miR-320a, and miR-423-5p have promising potential to serve as novel biomarkers for CRC detection, especially for early stage of CRC, which are superior to the currently used clinical biomarkers for CRC detection, such as CEA and CA19-9. Further efforts to develop the three microRNAs as biomarkers for early CRC diagnosis and prediction of surgical treatment outcomes are warrant.

**Electronic supplementary material:**

The online version of this article (doi:10.1186/s13046-015-0198-6) contains supplementary material, which is available to authorized users.

## Background

Colorectal carcinoma (CRC) is the one of the most common cancer-related mortalities worldwide [1]. In 2012, approximately 1,361,000 cases were diagnosed with CRC and 694,000 patients died of CRC according to the World Health Organization. Since early detection is critical for improving outcomes and reducing mortality of CRC patients, there is an urgent need to identify biomarkers for early diagnosis and prognosis of the disease. Currently, CEA and CA19-9 are the most commonly used circulating biomarkers for gastrointestinal tract tumor diagnosis [2]. However, the elevation of CEA concentration rarely occurs in patients with early stages CRC [2]. The sensitivity and validity of CEA detection are also not sufficient for early cancer detection [3]. The sensitivity of CA19-9 as a cancer biomarker is even less than that of CEA [4]. Except CEA and CA19-9, there are few other valuable biomarkers for monitoring CRC. Therefore, it is imperative to develop novel sensitive and specific circulating biomarkers for detection of CRC, especially in the early stages.

MicroRNAs are small non-coding RNAs of 18–22 nucleotides in length, which regulate gene expression at the post-transcriptional level by binding to the untranslated regions (UTRs) of mRNAs [5–7]. Since their discovery in 1993, emerging evidence shows that altered expression of microRNAs is associated with cancer, including CRC [1, 8–10]. MicroRNAs can be released to the blood. Since plasma microRNAs are protected from RNase digestions, they remain stable for a long period of time even under extreme harsh conditions. The stability and easy detectability make circulating microRNAs an ideal candidate to serve as a biomarker for cancer detection [11]. In addition, the abundance of plasma microRNAs normally does not vary in different gender [12–17]. Therefore, circulating microRNAs show great potential values as tumor markers for cancer diagnosis.

It has been reported that miR-24 and miR-320a act have tumor suppress activity in CRC. MiR-24 inhibits cell cycle progression via a p53- and p21-independent manner, and its expression is downregulated in CRC [18]. MiR-320a suppresses initiation, metastasis, and invasion of CRC [19–21]. In addition, it also induces G0/G1 arrest in CRC [22]. The expression of miR-320a is inversely associated with CRC aggressiveness of CRC and CRC cell lines [20]. Although expression of miR-423-5p, miR-24 and miR320a is down-regulated in CRC cell lines, it remains unknown whether plasma level of the three microRNA is changed in patients with CRC. To determine whether the abundance of miR-24, miR-320a, and miR435-5p in the plasma was changed in CRC patients and could serve as biomarkers for therapy evaluation, we measured the plasma concentration of miR-24, miR-320a, and miR435-5p in CRC patients prior or post the surgery, as well as healthy controls. The results showed that plasma levels of miR-24, miR-320a, and miR-423-5p were decreased in patients with CRC, benign lesions (polyps and adenoma) compared with healthy controls, but increased in patient with inflammatory bowel disease (IBD). The concentration of the three microRNAs was increased with the clinical improvement of the patients after the surgery. In addition, the three microRNAs all showed high detection rates for early stage of CRC. The results indicate that the plasma levels of miR-24, miR-320a, and miR-423-5p have the potential to serve as biomarkers for CRC detection, especially for early stage of CRC.

## Methods

### Ethics statement

The study was carried out according to the ethical principles of the 2008 revised Declaration of Helsinki. All plasma-based studies were approved by the Ethics Committee of the Xiamen University affiliated Zhongshan Hospital. All participants gave a written consent and agreed their information to be stored in the hospital database and used for research purposes.

### Plasma sample collection

Blood samples were collected from CRC patients who had been diagnosed categorized based on the International Union Against Cancer (UICC) and American Joint Committee on Cancer (AJCC) TNM staging system for CRC established in 2003. Age- and gender-matched healthy individuals with no history of cancer and in good health conditions based on self-report were collected from Xiamen University affiliated Zhongshan Hospital between December 2012 and November 2013. The IBD was diagnosed based on standard endoscopic, histologic, and radiographic criteria. Patients with other gastrointestinal tract complications, hemolysis, or high blood lipid were excluded. The blood samples were collected from patients before operational treatments, chemotherapy, or radiotherapy. At 10 days post the operation, paired plasma samples were collected from 43 randomly selected patients. All plasma samples were extracted from EDTA-K_2_ tubes and centrifuged as described previously [23]. After the first centrifugation for 10 min at 1,600 g, the supernatants were carefully removed and transferred to a new tube follow by centrifugation again at 16,000 g for 10 min to remove residual blood cells. The plasma was then divided into small aliquots and snap-frozen at −80 °C. Clinical characteristics of the CRC patients are summarized in Table 1.

**Table 1:** Clinicopathological characteristics of 111 CRC patients

Variables	n(%)
Gender	
Male	59(53.2 %)
Female	52(46.8 %)
Age(median, SD)	60 (14.23)
Location	
Rectun	54(48.6 %)
Colon	57(51.4 %)
T category	
T1	3(2.7 %)
T2	9(8.1 %)
T3	13(11.7 %)
T4	72 (64.9 %)
Unknown	14(12.6 %)
N category	
N0	55(49.6 %)
N1	42(37.8 %)
Unknown	14(12.6 %)
M category	
M0	89(80.2 %)
M1	22(19.8 %)
Tumor grade	
Stage I + II	54(48.7 %)
Stage III + IV	57(51.3 %)

### Biochemical analyses

The plasma concentration of CEA and CA19-9 was measured by using the Roche High-sensitivity Assay kit performed on a Cobas e601 System. The cut-off point of CEA is 5 ng/ml and the detection limit is 0.2 ng/ml with a CV of < 5 %. The cut-off point of CA19-9 is 27 U/ml and the detection limit is 0.6 U/ml with a CV of < 5 %. Samples were randomized for testing and blinded to the trained clinical laboratory technician who analyzed and interpreted the data.

### MicroRNA isolation

MicroRNA was isolated from 200 μl plasma samples using the miRcute miRNA extraction kit (TIANGEN) according to the manufacturer’s instructions. A synthetic microRNA cel-miR-39 (QIAGEN) was added to each plasma specimen at a final concentration of 5 nmol/ml as a reference before isolation. The purified microRNAs were dissolved in 30 μl RNase-free water (PROMEGA) at a concentration ranging from 5–50 ng/μl. The ratio of OD_260_ and OD_280_ absorbance of each sample was between 1.8 and 2.1. All isolated microRNAs were aliquoted and stored at a −80 °C freezer until use.

### cDNA synthesis

Reverse transcription (RT) was carried out in 20 μl solution that contained 0.2 μl 200 U/μl MMLV reverse transcriptase (PROMEGA), 0.2 μl 40 U/μl ribonuclease inhibitor (TAKARA), 0.8 μl 10 mmol/ml dNTP mix (TAKARA), 1.2 μl 10 mmol/ml stem-loop RT primer (SANGON), 4 μl MMLV RT buffer (PROMEGA), 11.6 μl RNAase-free water (PROMEGA), and 2 μl microRNA template. After being mixed gently, the reaction mixtures were incubated at 25 °C for 5 min, 40 °C for 60 min and then 70 °C for 15 min. The final cDNA products were stored at −20 °C until use. The reverse transcription primers are miR-24 [GenBank: AF480527.1], 5′-GTCGTATCCAGTGCGTGTCGTGGAGTCGGCAATTGCACTGATACGACCTGTTCCT-3′; miR-320a [GenBa-nk: JA682606.1], 5′-GTCGTATCCAGTGCGTGTCGTGGAGTCGGCAATTGCACTGGATACGACTCGCCCTC-3′; miR-423-5p [GenBank: JA830813.1], 5′-GTCGTATCCAGTGCGTGTCGTGGAGTCGGCAATTGCACTGGATACGACAAAGTCTC-3′; cel-miR-39 [GenBank: AJ487564.1], 5′-GTCGTATCCAGTGCGTGTCGTGGAGTCGGCAATTGCACTGGATACGACCAAGCTGA-3′.

### Quantitative real-time PCR

Real-time PCR was carried out following the manufacturer’s protocol of SYBR Premix Ex Taq TM II reagents (TAKARA) with 2 μl cDNA template. The PCR mixture (18 μl) contains 10 μl SYBR mix (TAKARA), 0.4 μl ROX Reference Dye II (TAKARA), 0.4 μl 10 mmol/ml forward primer (SANGON), 0.4 μl 10 mmol/ml reverse primer (SANGON) and 6.8 μl RNAase-free water (PROMEGA). PCR reaction was performed in duplicates. No-template PCR was used as a negative control. All real-time PCR reactions were carried out on an ABI7500 (ABI) machine. Reaction conditions were 95 °C for 30 s, followed by 40 cycles of 95 °C for 5 s and 60 °C for 34 s. The primers are: miR-24, 5′-GCAATGTGGCTCAGTTCAG-3′ (forward), 5′-CAGTGCGTGTCGTGGAGT-3′(reverse); miR-320a, 5′-AGGGCTAAAAGCTGGGTTGA-3′ (forward), 5′-CAGTGCGTGTCGTGGAGT-3′ (reverse); miR-423-5p, 5′-GCCTGAGGGGCAGAGAGC-3′ (forward), 5′-CCACGTGTCGTGGAGTC-3′ (reverse); cel-miR-39, 5′-CAGAGTCACCGGGTGTAAAT-3′ (forward), 5′-CCAGTGCGTGTCGTGGAGTC-3′ (reverse).

### Statistical analyses

Relative levels of the three microRNAs were quantified using the 2^-ΔΔCq^ method. The data then were transformed to log_10_ for analyses. The nonparametric Mann–Whitney *U* test and Kruskal–Wallis tests were used to analyze the abundance of miR-24, miR-320a and miR-423-5p in the disease and health groups. ROC curves were applied to analysis the diagnostic values of the three microRNAs. Youden index (sensitivity + specificity-1) was chosen to identify the optimal cut-off threshold values. Data were analyzed using two-side test and a *P* value < 0.05 was considered statistically significant. The statistical analyses were carried out with the IBM SPSS 19.0 software and the graphs were generated by using Graphpad Prism 5.0 and Canvas X.

## Results

### Establishing quantitative RT-PCR analyses for detecting miR-24, miR-320a, miR-423-5p and cel-miR-39 in plasma

Since abnormal expression of microRNAs are often associated with cancer progression, we identified the three microRNAs based on the following (1) up-regulated or down-regulated in CRC compared with adjacent tissues in the miRCancer database (http://mircancer.ecu.edu/search.jsp), or our previous study; (2) has not been analyzed in CRC patients plasma; and (3) Ct value in plasma is less than 35. A panel of 7 candidate microRNAs were selected for further analysis. For the training set, plasma samples of CRCs and healthy controls were randomly selected for qRT-PCR analyses. Among them, miR-24, miR-320a, and miR-423-5p were significantly down-regulated in CRC and were selected for the analyses hereafter.

The specific amplification and stability of the three candidate microRNAs in plasma was confirmed in Additional file 1. Melting curve analyses showed that there was only one unique peak for every sample (Additional file 1: Figure S1A-D). Agarose gel electrophoresis also showed one single band from randomly selected samples (Additional file 1: Figure S1E). The calculated slopes and coefficient of determination for miR-24 were −2.3815, r^2^ = 0.9992; miR-320a were −2.2526, r^2^ = 0.9974; miR-423-5p were −2.2247, r^2^ = 0.9964; and cel-miR-39 were −2.3732, r^2^ = 0.9998 (Additional file 1: Figure S1F), indicating that the amplification efficiencies for miR-24, miR-320a, miR-423-5p, and cel-miR-39 reached 98.15 %, 106.12 %, 107.89 %, and 98.59 %, respectively. Incubation of the samples at 37 °C for up to 24 h or repeating freeze-and-thaw did not cause significant changes in Cq value (Additional file 1: Figure S2), indicating that the microRNAs were stable in the plasma. Moreover, intra-assay variations and internal-assay variations were all less than 2 %, indicating that the RT-qPCR analyses were accurate and reliable (Additional file 1: Table S1 and S2). This warrants the RT-qPCR analyses of plasma miRNA for clinical applications.

### CRC patients have reduced abundances of miR-24, miR-320a, and miR-423-5p in the plasma

**Fig. 1:**
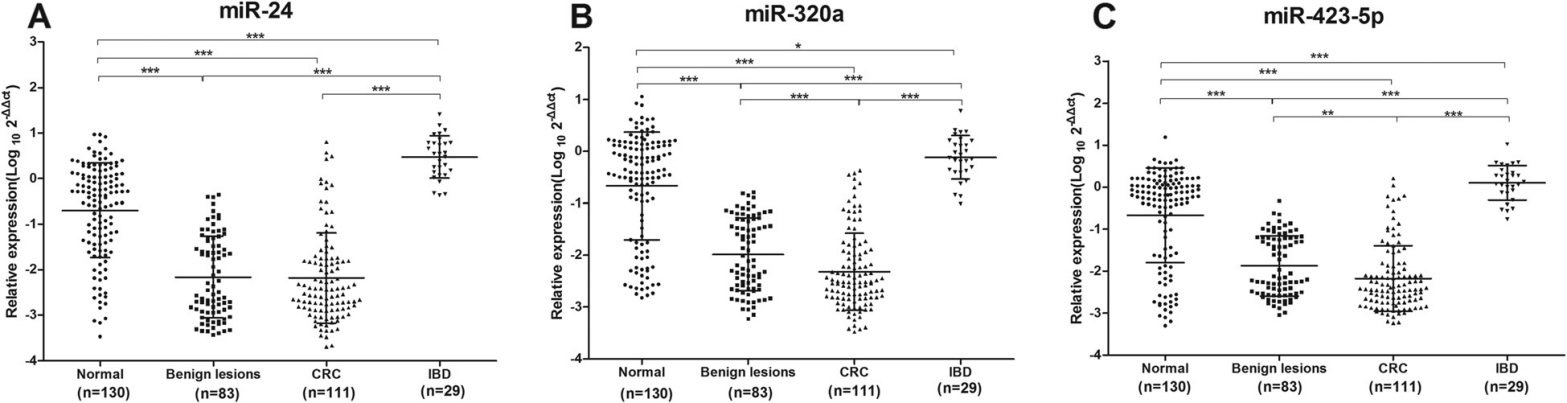
Plasma levels of miR-24, miR-320a, and miR-423-5p are reduced in patients with CRC. The relative levels of miR-24 (**a**), miR-320a (**b**) and miR-423-5p (**c**) in patients with benign lesions, CRC, IBD, and healthy controls. Numbers are normalized to the reference of cel-miR-39 and shown as log10. The Wilcoxon two-sample tests were performed to examine the difference of three microRNAs between normal controls and colorectal disease groups. (*, *p* < 0.05; **, *p*< 0.01; and *** *p* < 0.001)

To determine whether the plasma levels of miR-24, miR-320a, and miR-423-5p were changed in CRC patients, real-time RT-PCR was carried out to assess plasma concentrations of the three microRNAs in healthy controls and CRC patients. Compared with healthy controls, the abundances of all three microRNAs in the plasma were reduced in patients with CRC and benign lesions (colorectal adenoma and polyps), but increased in patients with IBD (Fig. 1). The expression levels of miR-320a and miR-423-5p were inversely correlated with the progression stages of the disease from normal-benign lesions-carcinoma. In addition, the plasma level of miR-320a and miR-423-5p was higher in patients with rectal cancer than those with CRC (Additional file 1: Figure S3A-C). No difference was found between patients with adenoma and polyps, as well as the patients with Crohn’s disease (CD) and with ulcerative colitis (UC) (Additional file 1: Figure S3D-I).

### The plasma levels of miR-24, miR-320a and miR-423-5p have diagnosis values for CRC

**Fig. 2:**
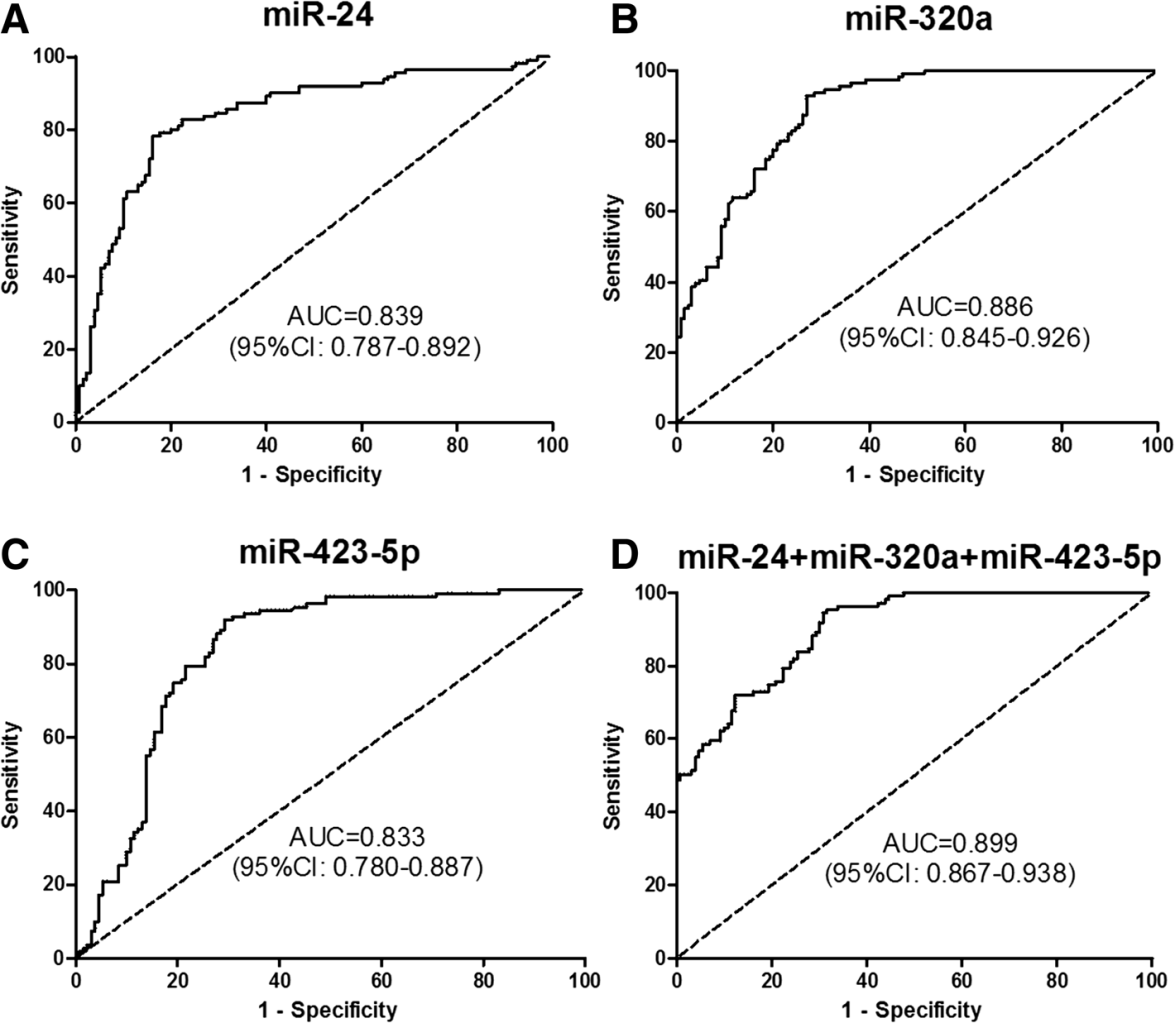
ROC curve analyses demonstrating that plasma levels of miR-24, miR-320a, and miR-423-5p are different among patients with CRC, benign lesions, and healthy controls. (**a-c**), ROC curves showing plasma levels of miR-24, miR-320a, and miR-423-5p in CRC patients are different from healthy controls. (**d**), Combination ROC curve analyses of the three microRNAs between CRC patients and healthy controls

To determine whether the plasma levels of miR-24, miR-320a, and miR-423-5p had CRC diagnostic values, the ROC curve was applied to analyze their diagnostic sensitivity and specificity (Fig. 2; Table 2). At the threshold of −1.731, the optimal sensitivity and specificity of miR-24 were 78.38 % and 83.85 % in distinguishing CRC from normal controls [area under curve (AUC) = 0.839]. At the threshold of −1.006, the sensitivity and specificity of miR-320a were 92.79 % and 73.08 % (AUC = 0.886). At the threshold of −0.854 (AUC = 0.833), and the sensitivity and specificity of miR-423-5p were 91.89 % and 70.77 %. Combined analyses of three microRNAs showed a higher AUC (0.899) and sensitivity (92.79 %) but with lower specificity (70.77 %) in distinguishing CRC patients and normal control. In order to determine whether the plasma level of the three microRNA had clinical values for CRC diagnosis, the PPV (positive predictive value), NPV (negative predictive value), and diagnosis efficiency were calculated. As shown in Table 2, the PPV for the three microRNAs were higher than 72 %. The results indicate that a person with high plasma levels of any one of the three microRNAs has a greater risk of CRC than those with low plasma levels of the three microRNAs. Additionally, the NPV and diagnosis efficiency of the three microRNAs were all higher than 80 %. The results indicate that the plasma levels of the three microRNAs have high diagnosis values.

**Table 2:** The diagnosis value between CRC and normal controls

Variables	miR-24	miR-320a	miR-423-5p	CEA	CA19-9
Cut-off	−1.731	−1.006	−0.854	5 ng/ml	27U/ml
Sensitivity	78.38 %	92.79 %	91.89 %	40.54 %	36.04 %
Specificity	83.85 %	73.08 %	70.77 %	95.38 %	93.08 %
PPV	80.56 %	74.64 %	72.86 %	88.24 %	81.63 %
NPV	81.95 %	92.23 %	82.88 %	65.26 %	63.02 %
Diagnosis efficiency	81.33 %	82.16 %	80.50 %	70.12 %	66.80 %

*PPV* positive predictive value, *NPV* negative predictive value

The AUC of miR-24 for distinguishing CRC and IBD was 0.9742 (95 % CI: 0.9513-0.9971) based on the optimal cut-off (−0.4363) according to Youden Index. The sensitivity and specificity was 91.89 % and 100 %, respectively. The AUC of miR-320a for distinguishing CRC and IBD was 0.9901(95 % CI: 0.9791-1.001). The sensitivity and specificity were 92.79 % and 100 % (cut-off: −1.025). At a threshold of −0.8093, the sensitivity and specificity of miR-423-5p were 91.89 % and 100 %.

### The plasma level of miR-24, miR-320a and miR-423-5p can be used for early detection of CRC

**Fig. 3:**
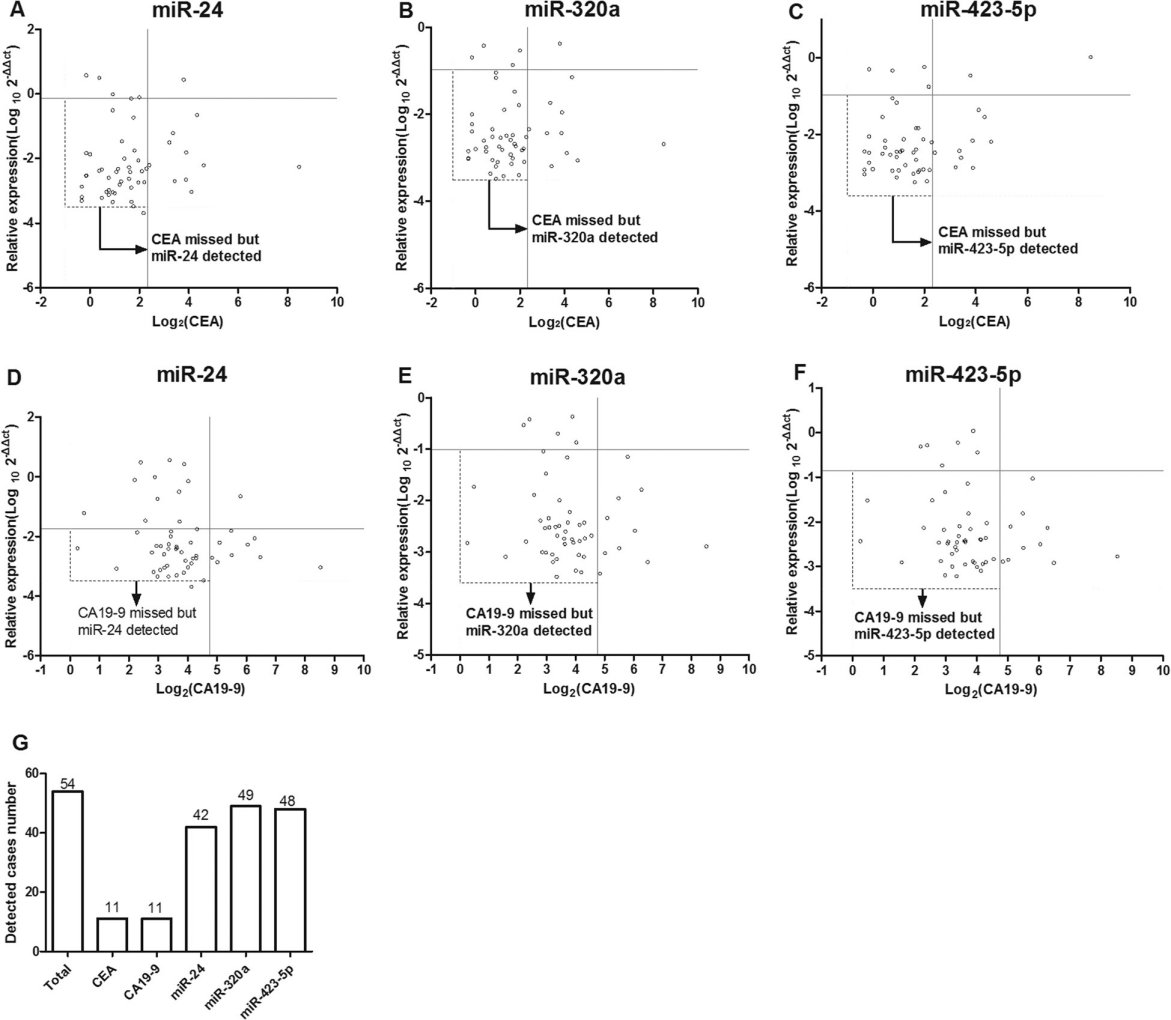
Plasma levels of miR-24, miR-320a, miR-423-5p are better biomarkers for early detection of CRC than currently used CEA, CA19-9. **a-f** Two-parameter classification is used to detect early stages of CRC. The cut-off value 5.0 ng/mL for CEA is, and 27U/ mL for CA19-9. The data is presented as log_2_. The cut-off values for miR-24, miR-320a and miR-423-5p are −1.731, −1.006, and −0.854, respectively. The data is calculated from the ROC curve. (**g**), Detection rates of the three microRNAs CEA, and CA19-9 in a total of 54 patients with early stages of CRC

**Fig. 4:**
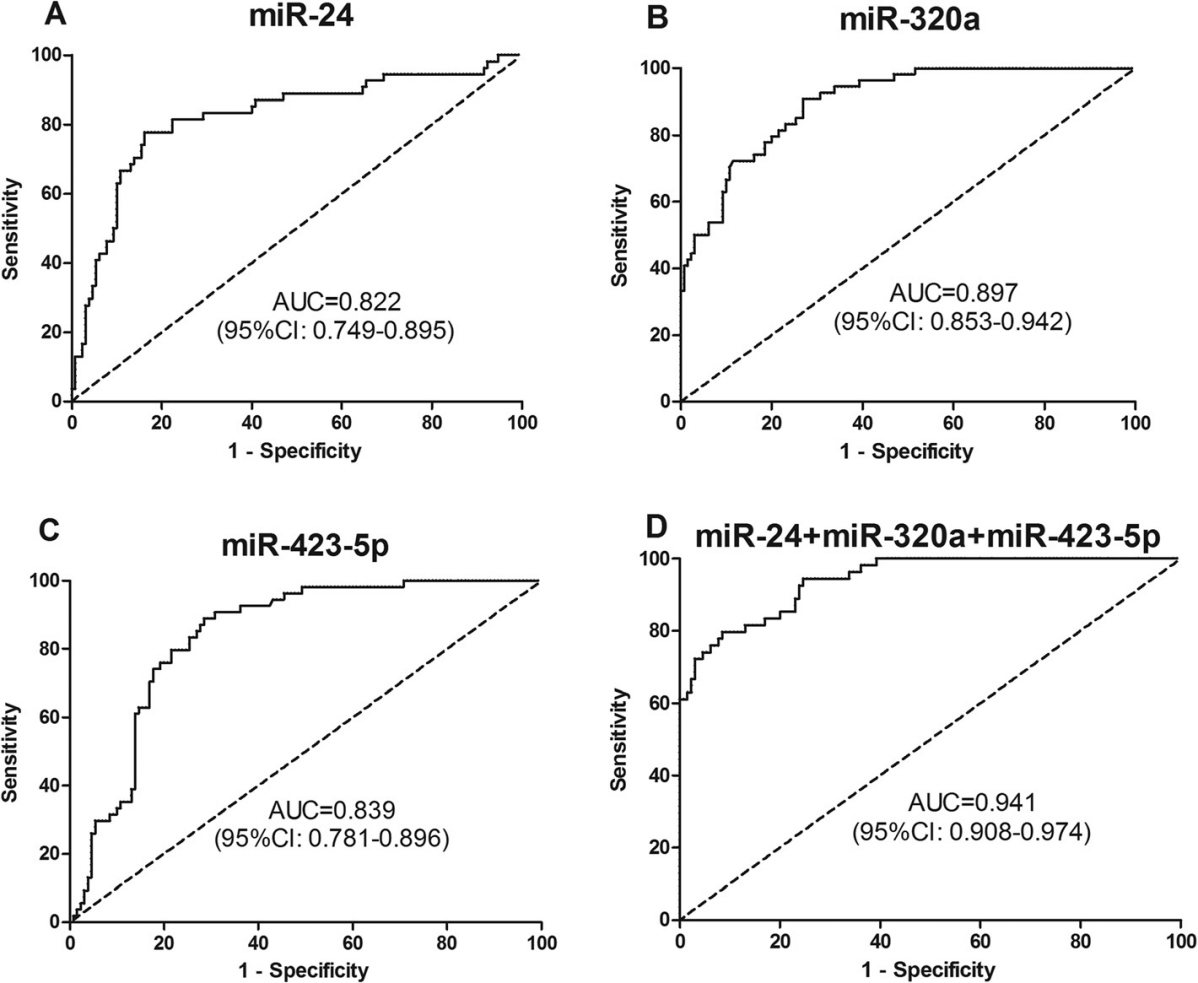
ROC curve analyses for detection of CRC at early stages (I, II). **a-c** ROC curves for miR-24, miR-320a, and miR-423-5p in 54 patients with early stages of and 130 healthy controls. **d** Combination ROC curve analyses of three microRNAs for patients with early stages of CRC and healthy controls

Early diagnosis and treatment of CRC is of great values to improve survival of CRC patients. Currently, CEA and CA19-9 are two most commonly used diagnosis markers for CRC. Therefore, the performance of the three microRNAs and CEA or CA19-9 in detecting early stages (stage I, stage II) of CRC was compared. In 54 patients with CRC at early stages, both CEA and CA19-9 were detected in 11 patients and the sensitivity was 20.37 %. In contrast, the plasma miR-24, miR-320a and miR-423-5p were detected in 42 (77.78 %), 49 (90.74 %), and 48 (88.89 %) patients, respectively (Fig. 3). According to the ROC curve, the AUC of three microRNAs reached 0.822, 0.897, and 0.839, respectively (Fig. 4a-c). By combining with the three microRNAs, the sensitivity was increased to 90.74 %, but the specificity dropped to 70.77 % (AUC = 0.941) (Fig. 4d, Table 3). Although the specificity of the three microRNAs was similar to CEA and CA19-9, the diagnosis efficiency and NPV, especially the sensitivity of the three microRNAs were higher than those of CEA and CA19-9. The findings validate the performance of miR-24, miR-320a, and miR-423-5p as a plasma marker for early detection of CRC, and indicate that the three microRNAs are better biomarkers for CRC early detection than CEA and CA19-9.

**Table 3:** The diagnosis value between early stage of CRC and normal controls

Variables	miR-24	miR-320a	miR-423-5p	CEA	CA19-9
Cut-off	−1.731	−1.006	−0.854	5 ng/ml	27U/ml
Sensitivity	77.78 %	90.74 %	88.89 %	20.37 %	20.37 %
Specificity	83.85 %	73.08 %	70.77 %	95.38 %	93.08 %
PPV	66.67 %	58.33 %	55.81 %	64.71 %	55 %
NPV	90.08 %	95 %	82.88 %	74.25 %	73.78 %
Diagnosis efficiency	82.07 %	78.26 %	76.09 %	73.37 %	71.74 %

### The changes in plasma level of miR-24, miR-320a and miR-423-5p after the surgery predicts the risk of post-surgery metastasis

**Fig. 5:**
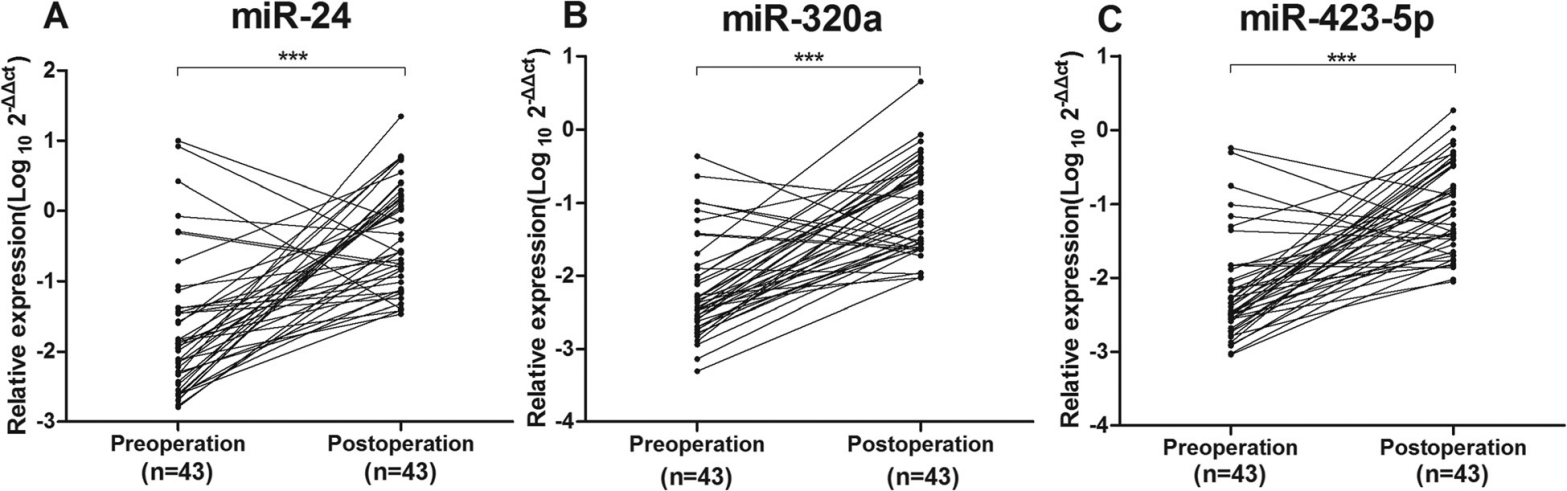
The changes of plasma levels of miR-24, miR-320a, and miR-423-5p before (Preoperation) and after surgery (Postoperation). The y-axis indicates the relative values of miR-24 (**a**), miR-320a (**b**) and miR-423-5p (**c**) which were normalized to cel-miR-39 and shown as log_10_ (***, *p* < 0.001, Wilcoxon matched-paired signed ranks sum test)

To determine whether the plasma levels of the three microRNAs had predicting values for clinical improvement after the surgery, the plasma levels of the three microRNAs in 43 patients were compared before and after the surgery. The data showed that the plasma levels of miR-24, miR-320a, and miR-423-5p in 37 (37/43 = 86.05 %), 35 (35/43 = 81.40 %), and 36 (36/43 = 83.72 %) patients, respectively, were increased after the surgery. The rest of the patients showed reduced plasma level of the three microRNAs after the surgery (Fig. 5). In the follow-up studies, it was found that the patients with reduced plasma level of the three microRNAs had high risk of liver metastasis (miR-24: 1/6 = 16.67 %; miR-320a, 1/8 = 12.50 %; miR-423-5p, 1/7 = 14.29 %) within six months later after the surgery. In contrast, the patients with increased plasma level of the three microRNAs had low risk of metastasis (miR-24: 1/31 = 3.2 %; miR-320a, 1/29 = 3.4 %; miR-423-5p, 1/30 = 3.3 %). Together, the data indicate that changes in plasma level of the three microRNAs after the surgery has prediction values for postoperative metastasis.

## Discussion

About 52.5 % microRNA coding sequences are located at fragile sites and genomic regions [24], and are more vulnerable to mutations or environmental influences. Aberrant microRNA expression and mutations have been shown to contribute to cancer initiation and progression [25–28], which can be potential biomarkers for cancer diagnosis and progression. Assessing microRNA expressions in CRC requires invasive examination, which limits the values of using microRNA as biomarkers for CRC diagnosis and prognosis. In our previous experiments, normal colorectal cells (CCD-18Co) have a higher expression than CRC cells (LS 174 T, HCT-8, SW480 and SW620) (Additional file 1: Figure S4). Herein, we report that expression of miR-24, miR-320a and miR-423-5p in the plasma of CRC patients was reduced and that changes of plasma levels of miR-24, miR-320a and miR-423-5p predicted the risk of post-surgery metastasis of CRC patients. The results indicate that the plasma level of miR-24, miR-320a and miR-423-5p can serve as normal biomarkers for CRC diagnosis and prognosis.

MiR-24 is a master regulator from the gene cluster of miR-23a–27a–24-2. Its expression is frequently down-regulated in a variety of cancers [18, 29–32], including CRC. It has been shown that miR-24 can function as a tumor suppressor in CRC, including suppressing proliferation, migration, and invation [18]. Moreover, miR-24 elicits its tumor suppression activity by repressing DHFR expression in CRC cells. MiR-320 is also a tumor suppressor. Expression of miR-320 is down-regulated in many human malignancies [33–35]. Overexpression of miR-320 inhibit colon cell proliferation, migration, and invasion. Moreover,miR-320 regulates the Wnt/β-catenin pathway partly by targeting FOXM1 that promotes the tumor initiation and progression [36]. In addition, miR-423-5p also contributes to proliferation and invasion in gastric cancer by targeting *TIF1* [37]. However, the role of miR-423-5p in CRC is largely unknown.

To our knowledge, this is the first comprehensively study on the expression and clinical significance of plasma levels of microRNAs-- miR-24, miR-320a, and miR-423-5p in CRC patients. Reduced plasma level of miR-24 was detected in 78.38 % patients with CRC, and that for miR-320a and miR-423-5p were even higher (>90 %). Moreover, all of the PPV were higher than 70 % and the diagnosis efficiency was higher than 80 %. Consistently, the AUCs for CEA and CA19-9 were both lower than three microRNAs whether in the diagnosis of CRC or early stages of CRC.

Clinical data shows that the 5-year survival rate of early stage of CRC patients after surgery is higher than 90 %. However, due to no obvious symptoms of early CRC and the lack of sensitive detection methods for early diagnosis, the majority of CRC patients are diagnosed at the advanced stages and lost their best time for treatment. Therefore, the low 5-year survival rate of post-operation is lower than 20 %. The colonoscopy can only detect 18 %–35 % of early CRC [38–40]. The two most commonly used biomarkers for CRC are CEA and CA19-9, which can only detecte 10 % and 15 % 1^st^ stage CRC, respectively [41]. Therefore, high sensitive biomarkers for CRC early detection are urgently needed. Detection of the plasma level of the three microRNAs significantly improve the detection rate of CRC at early stages (miR-24: 77.78 %; miR-320a: 90.74 %; miR-423-5p: 88.89 %). In conclusion, compared with CEA and CA19-9, the performance of the plasma level of the three microRNAs indicates that they have great clinical values for CRC detection.

The plasma levels of miR-24, miR-320a and miR-423-5p of patients with benign lesions (adenoma and polyps) were between those in normal controls and CRC patients. Furthermore, the expression of miR-320a and miR-423-5p was inversely correlated with the progression of the disease. This indicates that the plasma level miR-320a and miR-423-5p can be used to predict the disease conditions. Unlike patients with benign lesions (adenoma and polyps), the plasma levels of the three microRNAs were increased in patients with the IBD. The finding warrants the promise of using plasma levels of the three microRNAs as biomarkers for early detection of CRC, benign colorectal diseases, and IBD. Furthermore, the data also showed that the increased circulation levels of the three microRNAs were associated with the outcome of the surgical treatment. This suggests that the plasma levels of the three microRNAs are of potential prognosis values for CRC progression after the surgery.

## Conclusions

In summary, the plasma levels of miR-24, miR-320a, and miR-423-5p were reduced in patients with CRC and reversely correlated with the stages of progression. Furthermore, the changes of plasma level of the three microRNAs predicted the risk of post-surgery metastasis. The results suggest that the plasma level of miR-24, miR-320a, and miR-423-5p can serve as novel biomarkers for CRC diagnosis and prognosis.

## Additional file

Additional file 1:
**Figure S1.** Validation of the RT-qPCR analyses for cel-miR39, miR-24, miR-320a and miR-423-5p. Dissociation curves of miR-24 (A), miR-320a (B), miR-423-5p (C) and cel-miR-39 (D) were shown. (E), 2 % agarose gel electrophoresis of RT-qPCR production from randomly selected 2 plasma samples (M presents marker; 1, 2, 3, 4 present the product of miR-24, miR-320a, miR-423-5p and cel-miR-39, respectively, and B means blank control). (F), the calibration curves of miR-24, miR-320a, miR-423-5p and cel-miR-39, using serious 5 orders of magnitude dilution of cDNA were shown, and each point presents the average of duplication. **Figure S2.** The stability experiments of plasma miR-24, miR-320a and miR-423-5p. Plasma levels of miR-24, miR-320a, miR-423-5p were stable after prolonged 37 °C temperature incubation from 1 to 24 hours(A) or at least 5 freeze-thaw cycles(B). **Table S1.** Analysis of intra-assay variations. **Table S2.** Analysis of inter-assay variations. **Figure S3.** Plasma level of miR-24, miR-320a and miR-423-5p in different diseases groups. (A-C), the relative expression of three microRNAs in normal controls, patients with colon cancer and rectal cancer; (D-F), the relative level of three microRNAs in normal controls, patients with adenoma and polyps; (G-I), the relative abundance of three microRNAs in normal controls, patients with CD and UC; The Wilcoxon two-sample tests were performed to examine the difference of three microRNAs between each group. (**, p<0.01; ***, p<0.001; ns, non-significance). **Figure S4.** Relative expression of three candidate microRNAs in 4 CRC cell lines and 1 normal colorectal cell line. The relative quantification of miR-24 (A), miR-320a (B) and miR-423-5p (C) was standardized to reference gene U6. The bar chat was represented by mean ±SD (*, p<0.05).

## Abbreviations

miR-24: microRNA-24
miR-320a: microRNA-320a
miR-423-5p: microRNA-423-5p
CRC: Colorectal cancer
RT-qPCR: Reverse transcription quantitative real-time PCR
IBD: Inflammatory bowel disease
CEA: Carcino-embryonic antigen
CA19-9: Carbohydrate antigen19-9
UTRs: Untranslated regions
UICC: International Union Against Cancer
AJCC: American Joint Committee on Cancer
CV: Coefficient of variation
Cq: Quantification cycle
CD: Crohn’s disease
UC: Ulcerative colitis
ROC: Receiver operating characteristic curve
AUC: Area under curve

## References

[1] Zhang W, Zhang T, Jin R, Zhao H, Hu J, Feng B (2014). MicroRNA-301a promotes migration and invasion by targeting TGFBR2 in human colorectal cancer. J Exp Clin Cancer Res.

[2] Carpelan-Holmstrom M, Louhimo J, Stenman UH, Alfthan H, Haglund C (2002). CEA, CA 19–9 and CA 72–4 improve the diagnostic accuracy in gastrointestinal cancers. Anticancer Res.

[3] Locker GY, Hamilton S, Harris J, Jessup JM, Kemeny N, Macdonald JS (2006). ASCO 2006 update of recommendations for the use of tumor markers in gastrointestinal cancer. J Clin Oncol.

[4] Levin B, Lieberman DA, McFarland B, Andrews KS, Brooks D, Bond J (2008). Screening and surveillance for the early detection of colorectal cancer and adenomatous polyps, 2008: a joint guideline from the American Cancer Society, the US Multi-Society Task Force on Colorectal Cancer, and the American College of Radiology. Gastroenterology.

[5] Bartel DP (2004). MicroRNAs: genomics, biogenesis, mechanism, and function. Cell.

[6] Bartel DP (2009). MicroRNAs: target recognition and regulatory functions. Cell.

[7] Zhang M, Yang Q, Zhang L, Zhou S, Ye W, Yao Q (2014). miR-302b is a potential molecular marker of esophageal squamous cell carcinoma and functions as a tumor suppressor by targeting ErbB4. J Exp Clin Cancer Res.

[8] Liu M, Chen H (2010). The role of microRNAs in colorectal cancer. J Genet Genom.

[9] Dong Y, Wu WK, Wu CW, Sung JJ, Yu J, Ng SS (2011). MicroRNA dysregulation in colorectal cancer: a clinical perspective. Br J Cancer.

[10] Hrasovec S, Glavac D (2012). MicroRNAs as novel biomarkers in colorectal cancer. Front Genet.

[11] Ma R, Jiang T, Kang X (2012). Circulating microRNAs in cancer: origin, function and application. J Exp Clin Cancer Res.

[12] Lawrie CH, Gal S, Dunlop HM, Pushkaran B, Liggins AP, Pulford K (2008). Detection of elevated levels of tumour-associated microRNAs in serum of patients with diffuse large B-cell lymphoma. Br J Haematol.

[13] Mitchell PS, Parkin RK, Kroh EM, Fritz BR, Wyman SK, Pogosova-Agadjanyan EL (2008). Circulating microRNAs as stable blood-based markers for cancer detection. Proc Natl Acad Sci U S A.

[14] Chen X, Ba Y, Ma L, Cai X, Yin Y, Wang K (2008). Characterization of microRNAs in serum: a novel class of biomarkers for diagnosis of cancer and other diseases. Cell Res.

[15] Gilad S, Meiri E, Yogev Y, Benjamin S, Lebanony D, Yerushalmi N (2008). Serum microRNAs are promising novel biomarkers. PLoS One.

[16] Resnick KE, Alder H, Hagan JP, Richardson DL, Croce CM, Cohn DE (2009). The detection of differentially expressed microRNAs from the serum of ovarian cancer patients using a novel real-time PCR platform. Gynecol Oncol.

[17] Ng EK, Chong WW, Jin H, Lam EK, Shin VY, Yu J (2009). Differential expression of microRNAs in plasma of patients with colorectal cancer: a potential marker for colorectal cancer screening. Gut.

[18] Mishra PJ, Song B, Mishra PJ, Wang Y, Humeniuk R, Banerjee D (2009). MiR-24 tumor suppressor activity is regulated independent of p53 and through a target site polymorphism. PLoS One.

[19] Zhang Y, He X, Liu Y, Ye Y, Zhang H, He P (2012). microRNA-320a inhibits tumor invasion by targeting neuropilin 1 and is associated with liver metastasis in colorectal cancer. Oncol Rep.

[20] Sun JY, Huang Y, Li JP, Zhang X, Wang L, Meng YL (2012). MicroRNA-320a suppresses human colon cancer cell proliferation by directly targeting beta-catenin. Biochem Biophys Res Commun.

[21] Hsieh IS, Chang KC, Tsai YT, Ke JY, Lu PJ, Lee KH (2013). MicroRNA-320 suppresses the stem cell-like characteristics of prostate cancer cells by downregulating the Wnt/beta-catenin signaling pathway. Carcinogenesis.

[22] Zhao H, Dong T, Zhou H, Wang L, Huang A, Feng B (2014). miR-320a suppresses colorectal cancer progression by targeting Rac1. Carcinogenesis.

[23] Chim SS, Shing TK, Hung EC, Leung TY, Lau TK, Chiu RW (2008). Detection and characterization of placental microRNAs in maternal plasma. Clin Chem.

[24] Calin GA, Sevignani C, Dumitru CD, Hyslop T, Noch E, Yendamuri S (2004). Human microRNA genes are frequently located at fragile sites and genomic regions involved in cancers. Proc Natl Acad Sci U S A.

[25] Gao SM, Yang JJ, Chen CQ, Chen JJ, Ye LP, Wang LY (2012). Pure curcumin decreases the expression of WT1 by upregulation of miR-15a and miR-16-1 in leukemic cells. J Exp Clin Cancer Res.

[26] Zhou B, Chen H, Wei D, Kuang Y, Zhao X, Li G (2014). A novel miR-219-SMC4-JAK2/Stat3 regulatory pathway in human hepatocellular carcinoma. J Exp Clin Cancer Res.

[27] Liu J, Xue H, Zhang J, Suo T, Xiang Y, Zhang W (2015). MicroRNA-144 inhibits the metastasis of gastric cancer by targeting MET expression. J Exp Clin Cancer Res.

[28] Cheng Z, Wang HZ, Li X, Wu Z, Han Y, Li Y (2015). MicroRNA-184 inhibits cell proliferation and invasion, and specifically targets TNFAIP2 in Glioma. J Exp Clin Cancer Res.

[29] Volinia S, Calin GA, Liu CG, Ambs S, Cimmino A, Petrocca F (2006). A microRNA expression signature of human solid tumors defines cancer gene targets. Proc Natl Acad Sci U S A.

[30] Han ZB, Zhong L, Teng MJ, Fan JW, Tang HM, Wu JY (2012). Identification of recurrence-related microRNAs in hepatocellular carcinoma following liver transplantation. Mol Oncol.

[31] Xie Y, Tobin LA, Camps J, Wangsa D, Yang J, Rao M (2013). MicroRNA-24 regulates XIAP to reduce the apoptosis threshold in cancer cells. Oncogene.

[32] Gao Y, Liu Y, Du L, Li J, Qu A, Zhang X (2015). Down-regulation of miR-24-3p in colorectal cancer is associated with malignant behavior. Med Oncol.

[33] Cheng C, Chen ZQ, Shi XT (2014). MicroRNA-320 inhibits osteosarcoma cells proliferation by directly targeting fatty acid synthase. Tumour Biol.

[34] Wang X, Wu J, Lin Y, Zhu Y, Xu X, Xu X (2014). MicroRNA-320c inhibits tumorous behaviors of bladder cancer by targeting Cyclin-dependent kinase 6. J Exp Clin Cancer Res.

[35] Sun JY, Xiao WZ, Wang F, Wang YQ, Zhu YH, Wu YF (2015). MicroRNA-320 inhibits cell proliferation in glioma by targeting E2F1. Mol Med Rep.

[36] Wan LY, Deng J, Xiang XJ, Zhang L, Yu F, Chen J (2015). miR-320 enhances the sensitivity of human colon cancer cells to chemoradiotherapy in vitro by targeting FOXM1. Biochem Biophys Res Commun.

[37] Liu R, Zhang C, Hu Z, Li G, Wang C, Yang C (2011). A five-microRNA signature identified from genome-wide serum microRNA expression profiling serves as a fingerprint for gastric cancer diagnosis. Eur J Cancer.

[38] Shida H, Ban K, Matsumoto M, Masuda K, Imanari T, Machida T (1996). Asymptomatic colorectal cancer detected by screening. Dis Colon Rectum.

[39] Hardcastle JD, Chamberlain JO, Robinson MH, Moss SM, Amar SS, Balfour TW (1996). Randomised controlled trial of faecal-occult-blood screening for colorectal cancer. Lancet.

[40] Kitamura K, Taniguchi H, Yamaguchi T, Sawai K, Takahashi T (1997). Clinical outcome of surgical treatment for invasive early colorectal cancer in Japan. Hepatogastroenterology.

[41] Ogata-Kawata H, Izumiya M, Kurioka D, Honma Y, Yamada Y, Furuta K (2014). Circulating exosomal microRNAs as biomarkers of colon cancer. PLoS One.

