# Supplementary material for: Plasma levels of microRNA-24, microRNA-320a, and microRNA-423-5p are potential biomarkers for colorectal carcinoma
Source: J Exp Clin Cancer Res. 2015 Aug 22;34(1):86. doi: 10.1186/s13046-015-0198-6 (PMC4546358; doi:10.1186/s13046-015-0198-6)
Supplement: Additional file 1: — Figure S1. Validation of the RT-qPCR analyses for cel-miR39, miR-24, miR-320a and miR-423-5p. Dissociation curves of miR-24 (A), miR-320a (B), miR-423-5p (C) and cel-miR-39 (D) were shown. (E), 2 % agarose gel electrophoresis of RT-qPCR production from randomly selected 2 plasma samples (M presents marker; 1, 2, 3, 4 present the product of miR-24, miR-320a, miR-423-5p and cel-miR-39, respectively, and B means blank control). (F), the calibration curves of miR-24, miR-320a, miR-423-5p and cel-miR-39, using serious 5 orders of magnitude dilution of cDNA were shown, and each point presents the average of duplication. Figure S2. The stability experiments of plasma miR-24, miR-320a and miR-423-5p. Plasma levels of miR-24, miR-320a, miR-423-5p were stable after prolonged 37 °C temperature incubation from 1 to 24 hours(A) or at least 5 freeze-thaw cycles(B). Table S1. Analysis of intra-assay variations. Table S2. Analysis of inter-assay variations. Figure S3. Plasma level of miR-24, miR-320a and miR-423-5p in different diseases groups. (A-C), the relative expression of three microRNAs in normal controls, patients with colon cancer and rectal cancer; (D-F), the relative level of three microRNAs in normal controls, patients with adenoma and polyps; (G-I), the relative abundance of three microRNAs in normal controls, patients with CD and UC; The Wilcoxon two-sample tests were performed to examine the difference of three microRNAs between each group. (**, p<0.01; ***, p<0.001; ns, non-significance). Figure S4. Relative expression of three candidate microRNAs in 4 CRC cell lines and 1 normal colorectal cell line. The relative quantification of miR-24 (A), miR-320a (B) and miR-423-5p (C) was standardized to reference gene U6. The bar chat was represented by mean ±SD (*, p<0.05). [file 13046_2015_198_MOESM1_ESM.docx]

**Additional file 1**

**
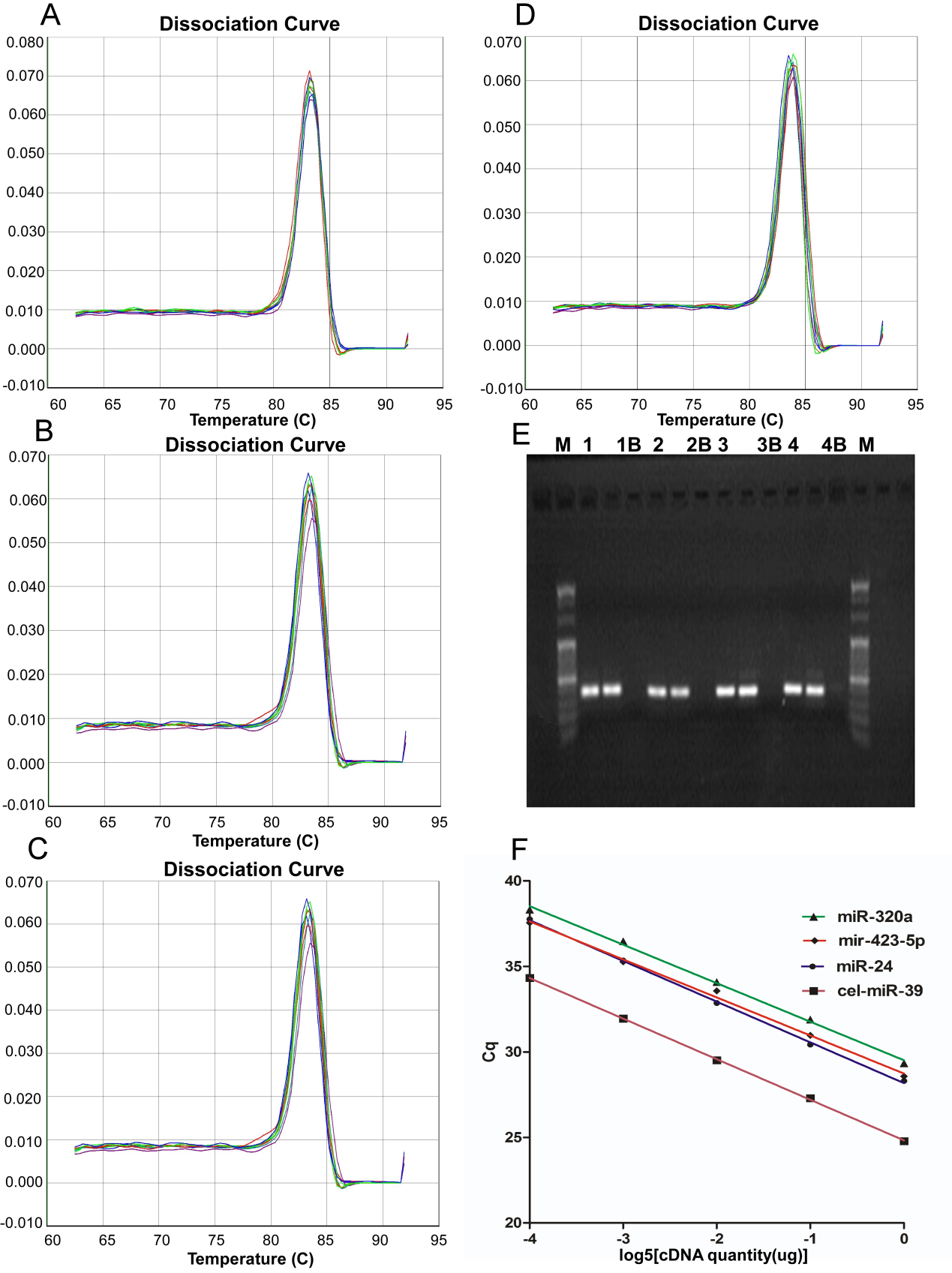
**

**Figure S1. Validation of the RT-qPCR analyses for for miR-24, miR-320a, miR-423-5p and cel-miR-39**. Dissociation curves of miR-24 (A), miR-320a (B), miR-423-5p (C), and cel-miR-39 (D) were shown. (E), 2% agarose gel electrophoresis of RT-qPCR products of randomly selected 2 plasma samples. M molecular weight markers; 1-4, PCR products of miR-24, miR-320a, miR-423-5p, and cel-miR-39, respectively; B, blank control without templates. (F), the calibration curves of miR-24, miR-320a, miR-423-5p, and cel-miR-39, using serious 5 orders of magnitude dilution of cDNAs. Each point represents the average of duplication.





**Figure S2. MiR-24, miR-320a and miR-423-5p in the plasma are stable.** Real-time RT-PCR analyses of miR-24, miR-320a, and miR-423-5p in the plasma after incubation at 37℃ from 1 to 24 hours (A) or subjected to 1-5 freeze-thaw cycles (B).

**Table S1 Analysis of intra-assay variations**

**Samples miR-24 miR-320a miR-423-5p cel-miR-39**

**mean±SD CV(%) mean±SD CV(%) mean±SD CV(%) mean±SD CV(%)**

Sample 1 26.73±0.13 0.48% 29.33±0.25 0.86% 26.77±0.03 0.11% 24.95±0.07 0.27%

Sample 2 26.21±0.06 0.22% 29.37±0.11 0.36% 26.79±0.07 0.26% 24.97±0.23 0.92%

Sample 3 27.78±0.08 0.28% 28.96±0.18 0.62% 27.57±0.05 0.16% 25.51±0.20 0.80%

Sample 4 31.43±0.45 1.42% 29.95±0.13 0.43% 29.71±0.07 0.25% 27.67±0.06 0.22%

Sample 5 26.05±0.02 0.07% 27.79±0.05 0.20% 25.59±0.14 0.53% 25.13±0.21 0.83%

Sample 6 26.89±0.18 0.69% 29.44±0.22 0.73% 26.80±0.18 0.66% 26.14±0.05 0.19%

Sample 7 28.79±0.45 1.55% 30.05±0.15 0.51% 28.85±0.25 0.85% 27.45±0.37 1.34%

Sample 8 25.09±0.17 0.68% 28.41±0.22 0.78% 25.20±0.28 1.11% 26.74±0.10 0.37%

**Table S2 Analysis of inter-assay variations**

**Samples miR-24 miR-320a miR-423-5p cel-miR-39**

**mean±SD CV(%) mean±SD CV(%) mean±SD CV(%) mean±SD CV(%)**

Sample 1 26.66±0.21 0.80% 29.45±0.21 0.71% 27.46±0.42 1.54% 25.23±0.16 0.62%

Sample 2 26.33±0.25 0.93% 29.58±0.16 0.55% 27.36±0.32 1.18% 25.22±0.31 1.21%

Sample 3 27.84±0.37 1.32% 29.13±0.14 0.49% 28.12±0.31 1.11% 25.79±0.20 0.76%

Sample 4 31.28±0.18 0.60% 30.18±0.13 0.44% 30.34±0.39 1.29% 27.92±0.18 0.64%

Sample 5 26.02±0.18 0.70% 27.97±0.16 0.57% 26.14±0.34 1.31% 25.54±0.30 1.17%

Sample 6 26.91±0.16 0.58% 29.59±0.11 0.38% 27.42±0.37 1.37% 26.23±0.30 1.13%

Sample 7 28.69±0.23 0.81% 30.34±0.20 0.66% 29.33±0.32 1.08% 27.34±0.33 1.22%

Sample 8 25.10±0.17 0.66% 28.59±0.18 0.62% 26.03±0.47 1.80% 27.12±0.46 1.70%

**
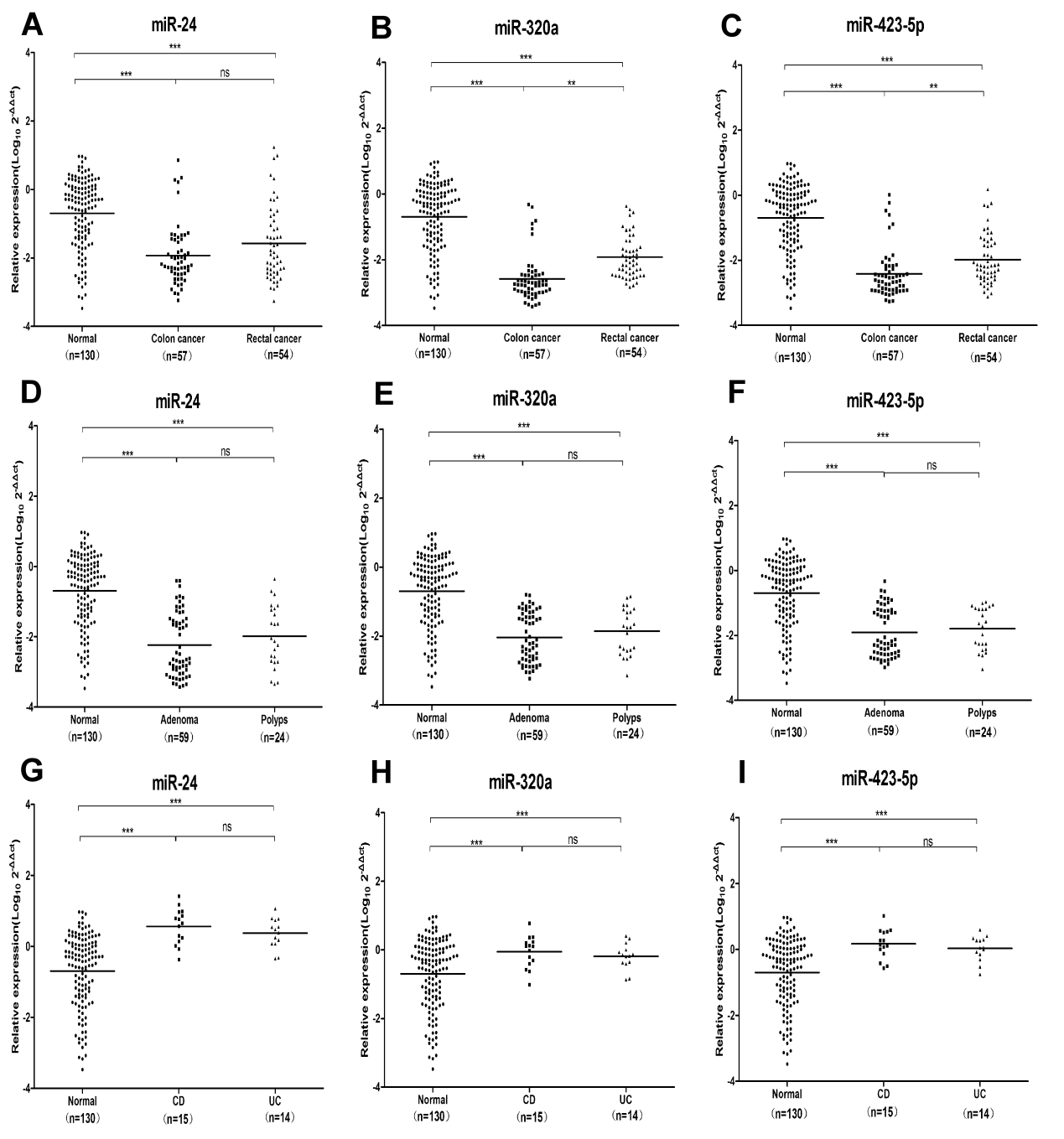
**

**Figure S3. CRC patients have reduced plasma level of miR-24, miR-320a, and miR-423-5p.** (A-C). Real-time RT-PCR analyses of the three microRNAs in normal controls, patients with colon or rectal cancer. (D-F), Relative level of the three microRNAs in normal controls, patients with colon adenoma and polyps. (G-I), Relative abundance of the three microRNAs in normal controls, patients with CD and UC. The Wilcoxon two-sample tests were performed to examine the difference of plasma levels of the three microRNAs between individual groups. (**, *p*<0.01; ***, *p*<0.001; ns, non-significance).


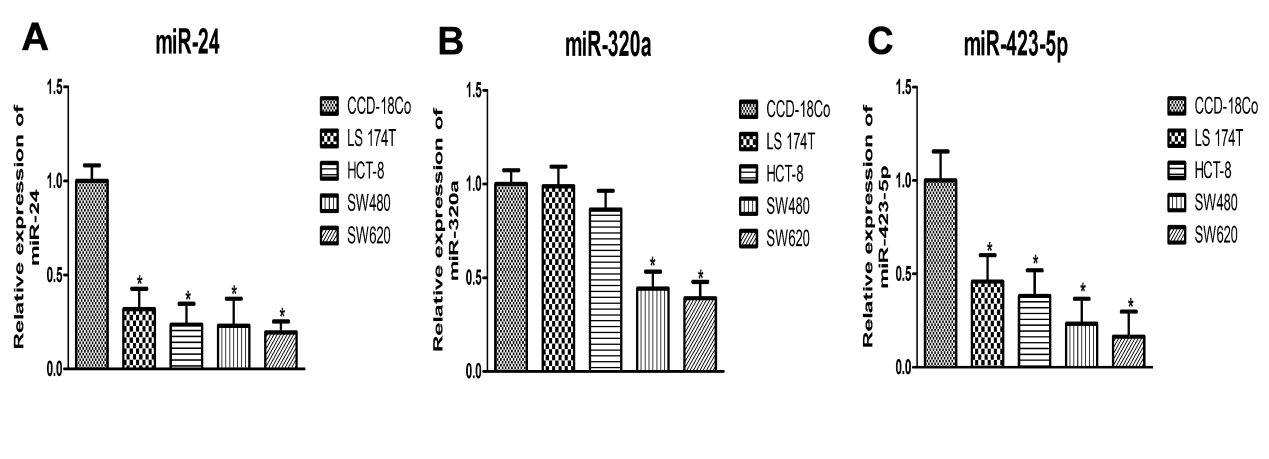


**Figure S4. Expression of miR-24, miR-320a, and miR-423-5p is reduced in CRC cells.** Real-time RT-PCR analyses of miR-24 (A), miR-320a (B), and miR-423-5p (C) in the indicated cell lines. Data was standardized to reference gene U6 and are expressed as mean ±SD (*, *p*<0.05).
